# DPEP1 Inhibits Tumor Cell Invasiveness, Enhances Chemosensitivity and Predicts Clinical Outcome in Pancreatic Ductal Adenocarcinoma

**DOI:** 10.1371/journal.pone.0031507

**Published:** 2012-02-20

**Authors:** Geng Zhang, Aaron Schetter, Peijun He, Naotake Funamizu, Jochen Gaedcke, B. Michael Ghadimi, Thomas Ried, Raffit Hassan, Harris G. Yfantis, Dong H. Lee, Curtis Lacy, Anirban Maitra, Nader Hanna, H. Richard Alexander, S. Perwez Hussain

**Affiliations:** 1 Laboratory of Human Carcinogenesis, Center for Cancer Research, National Cancer Institute, National Institutes of Health (NIH), Bethesda, Maryland, United States of America; 2 Department of General and Visceral Surgery, University Medicine, Göttingen, Germany; 3 Genetics Branch, National Cancer Institute, NIH, Bethesda, Maryland, United States of America; 4 Laboratory of Molecular Biology, National Cancer Institute, NIH, Bethesda, Maryland, United States of America; 5 Pathology and Laboratory Medicine, Baltimore Veterans Affairs Medical Center, Baltimore, Maryland, United States of America; 6 The Sol Goldman Pancreatic Cancer Research Center, School of Medicine, Johns Hopkins University, Baltimore, Maryland, United States of America; 7 Division of Surgical Oncology, The Department of Surgery and the Marlene, School of Medicine, Stewart Greenebaum Cancer Center, University of Maryland, Baltimore, Maryland, United States of America; Vanderbilt University Medical Center, United States of America

## Abstract

Pancreatic ductal adenocarcinoma (PDAC) is one of the most lethal cancers worldwide. To identify biologically relevant genes with prognostic and therapeutic significance in PDAC, we first performed the microarray gene-expression profiling in 45 matching pairs of tumor and adjacent non-tumor tissues from resected PDAC cases. We identified 36 genes that were associated with patient outcome and also differentially expressed in tumors as compared with adjacent non-tumor tissues in microarray analysis. Further evaluation in an independent validation cohort (N = 27) confirmed that *DPEP1* (dipeptidase 1) expression was decreased (T: N ratio ∼0.1, P<0.01) in tumors as compared with non-tumor tissues. DPEP1 gene expression was negatively correlated with histological grade (Spearman correlation coefficient = −0.35, *P* = 0.004). Lower expression of DPEP1 in tumors was associated with poor survival (Kaplan Meier log rank) in both test cohort (*P* = 0.035) and validation cohort (*P* = 0.016). DPEP1 expression was independently associated with cancer-specific mortality when adjusted for tumor stage and resection margin status in both univariate (hazard ratio = 0.43, 95%CI = 0.24–0.76, P = 0.004) and multivariate analyses (hazard ratio = 0.51, 95%CI = 0.27–0.94, P = 0.032). We further demonstrated that overexpression of DPEP1 suppressed tumor cells invasiveness and increased sensitivity to chemotherapeutic agent Gemcitabine. Our data also showed that growth factor EGF treatment decreased DPEP1 expression and MEK1/2 inhibitor AZD6244 increased DPEP1 expression *in vitro*, indicating a potential mechanism for *DPEP1* gene regulation. Therefore, we provide evidence that DPEP1 plays a role in pancreatic cancer aggressiveness and predicts outcome in patients with resected PDAC. In view of these findings, we propose that DPEP1 may be a candidate target in PDAC for designing improved treatments.

## Introduction

PDAC is the fourth leading cause of cancer deaths in the United States and is among the most lethal human malignancies worldwide with a median survival of 6 months and 5-year survival of 6% [1]. An estimated 44,030 new cases and 37,660 deaths are expected to occur in the United States in 2011(American Cancer Society, Cancer Facts & Figures 2011). The dismal outcome in pancreatic cancer patients is attributed to the late diagnosis and resistance to the available chemotherapy. In less than 20% of the patients, surgical resection is an option with some potential for cure. The median survival even for resected patients is less than 2 years with recurrence in ∼80% of the cases within this time period. However, about 12% of the resected patients may survive for 5 years, which is attributed not only to the stage, grade and resection margin status but also to the distinct biological makeup of tumors [2], [3].

Gemcitabine is the first-line chemotherapeutic drug commonly used for advanced pancreatic cancer. However, single agent gemcitabine is only moderately effective with a tumor response rate of ∼12% [4]. In a recent phase 3 trial, FOLFIRINOX regimen (oxaliplatin, irinotecan, fluorouracil and leucovorin) significantly enhanced median survival as compared to gemcitabine (11.1 vs. 6.8 months, P<0.001), in patients with metastatic pancreatic cancer [5]. Despite recent progress in chemotherapy, better understanding of molecular mechanism of this disease and discovery of novel therapeutic targets are desperately needed to improve outcomes in patients with PDAC.

One strategy to identify the potential targets for pancreatic cancer treatments is to distinguish and investigate genes and pathways that are associated with patient outcome and biologically relevant to the aggressiveness of PDAC [2]. Gene-expression profiling using microarrays has been utilized to identify genes or gene signatures that are associated with pancreatic cancer [6], [7], [8]. A few studies have defined and validated gene signatures that are associated with survival, pathological stage and metastasis using microarrays, providing insight into molecular subtypes of PDAC and revealing several promising targets for cancer treatment [9], [10], [11]. In the present study, we first identified genes that were associated with cancer-specific mortality by microarray gene expression analysis and validated them by quantitative RT-PCR in two independent cohorts of resected PDAC cases. We then explored the biological function of DPEP1, a predictor of patient outcome identified in our study, revealing its potential therapeutic significance in pancreatic cancer.

## Materials and Methods

### Tissue Collection

Matched pairs of primary pancreatic tumor and adjacent non-tumor tissues came from 45 patients with PDCA at the University of Medicine, Göttingen, Germany, and from 27 patients recruited from the University of Maryland Medical System (UMMS) at Baltimore, Maryland through NCI-UMD resource contract. Tissues were flash frozen immediately after surgery. Demographic and clinical information for each tissue donor, including age, sex, clinical staging, resection margin status, survival times from diagnosis, and receipt of adjuvant chemotherapy were collected. Tumor histopathology was classified according to the World Health Organization Classification of Tumor system [12]. Use of these clinical specimens was reviewed by the NCI-Office of the Human Subject Research (OHSR, Exempt # 4678) at the National Institutes of Health, Bethesda, MD.

### RNA Isolation and Microarray Processing

RNA from frozen tissue samples was extracted using standard TRIZOL (Invitrogen) protocol. RNA quality was confirmed with the Agilent 2100 Bioanalyzer (Agilent Technologies) before the microarray gene expression profiling. Tumors and paired non-tumor tissues from Germany cohort were profiled using the Affymetrix GeneChip Human 1.0 ST arrays according to the manufacturer's protocol at LMT microarray core facility at National Cancer Institute, Frederick, MD. All arrays were RMA normalized and gene expression summaries were created for each gene by averaging all probe sets for each gene using Partek Genomics Suite 6.5. All data analysis was performed on gene summarized data. The microarray gene expression data has been deposited in the National Center for Biotechnology Information's (NCBIs) Gene Expression Omnibus (GEO; http://www.ncbi.nlm.nih.gov/geo) with accession numbers GSE28735.

### Quantitative RT-PCR (qRT-PCR)

Total RNA was reverse transcribed using High-Capacity cDNA Reverse Transcription Kit (Applied Biosystems). High-throughput qRT-PCR of gene expression was performed using 96.96 dynamic array chips from Fluidigm Corporation according to the manufacturer's protocol. Pre-amplification reactions were done in a GeneAmp PCR System 9700 from Applied Biosystems. The IFC Controller HX (Fluidigm Corporation) utilizes pressure to control the valves in the chips and load samples and gene expression assay reagents into the reaction chambers. The BioMark system (Fluidigm Corporation) is a real-time PCR instrument designed to thermal cycle these microfluidic chips and image the data in real time. qRT-PCR reactions in 384 well plates were performed using Taqman Gene Expression Assays on an ABI Prism 7900HT Sequence Detection instrument from Applied Biosystems. Expression levels of GAPDH were used as the endogenous controls. All assays were performed in quadruplates or triplicates. For quality control, any samples with a gene cycle value greater than 36 were considered of poor quality and removed. If a tumor or non-tumor sample failed quality control from qRT-PCR that case was removed from the analysis. All the primers for qRT-PCR in the present study were purchased from Applied Biosystems (Table S1).

### Statistical Analysis

T-test, Wilcoxon matched-pairs t-tests, and expression graphs were used to analyze differences in gene expression between tumors and paired non-tumor tissues using Graphpad Prism 5.0 (Graphpad Software Inc, San Diego, California). Correlation analysis and Kaplan-Meier analysis were performed with Graphpad Prism 5.0. Fisher's exact test and Cox Proportional-hazards regression analysis were performed using Stata 11 (StataCorp LP, College Station, Texas). Univariate Cox regression was performed on genes and clinical covariates to examine influence of each on patient survival. Final multivariate models were based on stepwise addition and removal of clinical covariates found to be associated with survival in univariate models (*P*<0.05). For these models, resection margin status was dichotomized as positive (R1) vs negative (R0); TNM staging was dichotomized based on non-metastatic (I-IIA) vs metastatic (IIB-IV) disease; histological grade was dichotomized based on well and moderate differentiation (G1&2) vs poorly differentiation(G3&4). All stepwise addition models gave the same final models as stepwise removal models. All univariate and multivariate Cox regression models were tested for proportional hazards assumptions based on Schoenfeld residuals, and no model violated these assumptions. The statistical significance was defined as *P*<0.05. All *P* values reported were 2- sided.

### Cell lines and culture conditions

Human pancreatic carcinoma cell lines Panc1 (CRL-1469™) and MIApaca2 (CRL-1420™), were obtained from American Type Culture Collection ATCC (Rockville, MD, USA). Cells were maintained in GIBCO® RPMI Media 1640 supplemented with GlutaMAX™-I (Invitrogen), penicillin-streptomycin (50 IU/ml and 50 mg/ml, respectively), and 10% (v/v) fetal calf serum (FCS). LY294002 was purchased from Cell Signaling and dissolved in DMSO (dimethyl sulfoxide) to make 50 mM stock solution. AZD6244 were purchased from ChemieTek and dissolved in DMSO to make 40 mM stock solutions. Human EGF was purchased from BD bioscience and reconstituted to make 100 mM stock. Cells were incubated at 37°C in a humidified atmosphere with 10% CO_2_. Transfection of the plasmid was performed by using Lipofectamine LTX reagent according to the manufacturer's protocol (Invitrogen, USA). Plasmid pCMV-GFP was used for transfection as the negative control. Plasmid pCMV-DPEP1-GFP was used for DPEP1-overexpression.

### MTT assay

Cells were seeded in 96-well plates (3,000 cells/well) and incubated for 2–10 days. Then, the MTT solution was added and incubated for 4 hours. The solution was aspirated and 100 ul DMSO was added to each well. The absorbance was measured at 570 nm and 650 nm.

### Cell invasion assay

Pancreatic cancer cell invasion assay was performed in 24-well Biocoat Matrigel invasion chambers (8 µm; Becton Dickinson) according to the manufacturer's protocol. Briefly, cells were transfected with either control vector pCMV-GFP or pCMV-DPEP1-GFP expression vectors respectively. 24 h following transfection, cells were harvested and plated in the top chamber (5×10^4^/well). The bottom chamber contained 10% FBS as a chemoattractant. After 48 h incubation, the noninvasive cells were removed with a cotton swab. The invasive cells had migrated through the membrane and stuck to the lower surface of the membrane. GFP-positive cells were counted under a fluorescence microscope in five fields (20× magnification). The result is expressed as the percent invasion according to the manufacturer's manual: the number of the cells invading through the Matrigel membrane divided by the number of cells migrating through the control membrane. All assays were performed in triplicate.

### Drug cytotoxicity assay

Gemcitabine was purchased from Tocris Bioscience and dissolved in DPBS (Dulbecco's Phosphate-Buffered Saline) to make 20 mM stock solution. Cells were seeded into 96-well plates at 10,000 cells per well, allowed to grow for 24 hours, and then treated with gemcitabine for another 96 hours. Different concentrations of gemcitabine were made in serum-free medium. At the end of treatment, cell viability was determined by the CellTiter-Glo Assay (Promega).

## Results

### Gene expression microarray analysis to identify candidate genes associated with patient outcome

The characteristics of the patients with PDAC in the test cohort (N = 45, from Germany) and the validation cohort (N = 27, from Maryland) are shown in Table 1. The two cohorts were similar in TNM staging, resection margin status, and cancer-specific mortality (*P* = 0.44, Kaplan-Meier log rank) with 1-year survival rate of 64.5% for the Germany cohort and 63.0% for the Maryland cohort. The Germany cohort was older than the Maryland cohort (average 68.4 vs 61.9 years).

**Table 1:** Characteristics of population.

	Germany cohort^a^ (n = 45)	Maryland cohort^b^(n = 27)	Combined cohort (n = 72)	*P*
Age at enrollments (y)				0.003^c^
Mean (SD)	68.4 (7.5)	61.9 (10.6)	65.9 (9.5)	
Range	47–83	38–82	38–83	
Gender, no. (%)				0.63^d^
Male	21 (47)	15 (56)	36 (50)	
Female	24 (53)	12 (44)	36 (50)	
Resection margin, no. (%)				0.46^d^
R1	23 (51)	17 (63)	40 (56)	
R0	22 (49)	10 (37)	32 (44)	
TNM stage, no. (%)				0.109^d^
I	1 (2)	4 (15)	5 (7)	
IIA	6 (14)	4 (15)	10 (15)	
IIB	21 (50)	15 (58)	36 (53)	
III	9 (21)	1 (4)	10 (15)	
IV	5 (12)	2 (8)	7 (10)	
Grading, no. (%)				0.33^d^
G1&2	22 (49)	17 (63)	39 (54)	
G3&4	23 (51)	10 (37)	33 (46)	

a The survival information of 3 patients was not available in German cohort, so these 3 patients were removed from analyses.

b For one patient with liver metastasis in the Maryland cohort, the exact stage of this patient is unclear, so this patient was removed from Fisher's exact test. Because this patient already had liver metastasis, the tumor stage was treated as over IIB in the Cox regression.

c T-test for comparison of Gemany and Maryland cohorts.

d Fisher's exact test for comparison of Gemany and Maryland cohorts.

We compared gene expression profile of 45 pairs of pancreatic tumor and adjacent non-tumor tissues in the Germany cohort using Affymetrix GeneChip Human 1.0 ST arrays. Tumor gene expression profiles were distinctly different from non-tumor profiles. Using ANOVA, 2620 genes were found to be differentially expressed in tumors as compared to non-tumor (*P*<0.001). We next performed Cox-regression analysis and identified 277 of the differentially expressed genes that were associated with survival (*P*<0.05). The list of 277 genes was then subjected to pathway and biomarker analyses using Ingenuity Pathways Analysis (IPA), and 36 genes were selected based on their potential association with cancer-related pathways for further analyses (Table S1, Figure S1A).

### DPEP1 and TPX2 are associated with cancer-specific mortality in both test and validation cohorts

We further confirmed the expression levels of these 36 genes with qRT-PCR in tumor and non-tumor tissues in the Germany test cohort. The microarray gene expression data and qRT-PCR data were highly correlated (Spearman r = 0.89, *P*<0.0001, Figure.S1B). qRT-PCR results showed that 25 genes were differentially expressed in the test cohort (*P*<0.01, Table S1). The top differentially expressed transcripts between tumor and non-tumor were *TPX2*, *DCBLD2* and *ANLN* which were significantly increased in tumors (T∶N ratio>2.0, *P*<0.01), and *CDO1*, *DPEP1*, *C7*, *ALDH1A1* and *NR3C2* which were significantly decreased in tumors (T∶N ratio<0.2, *P*<0.01).

**Figure 1:**
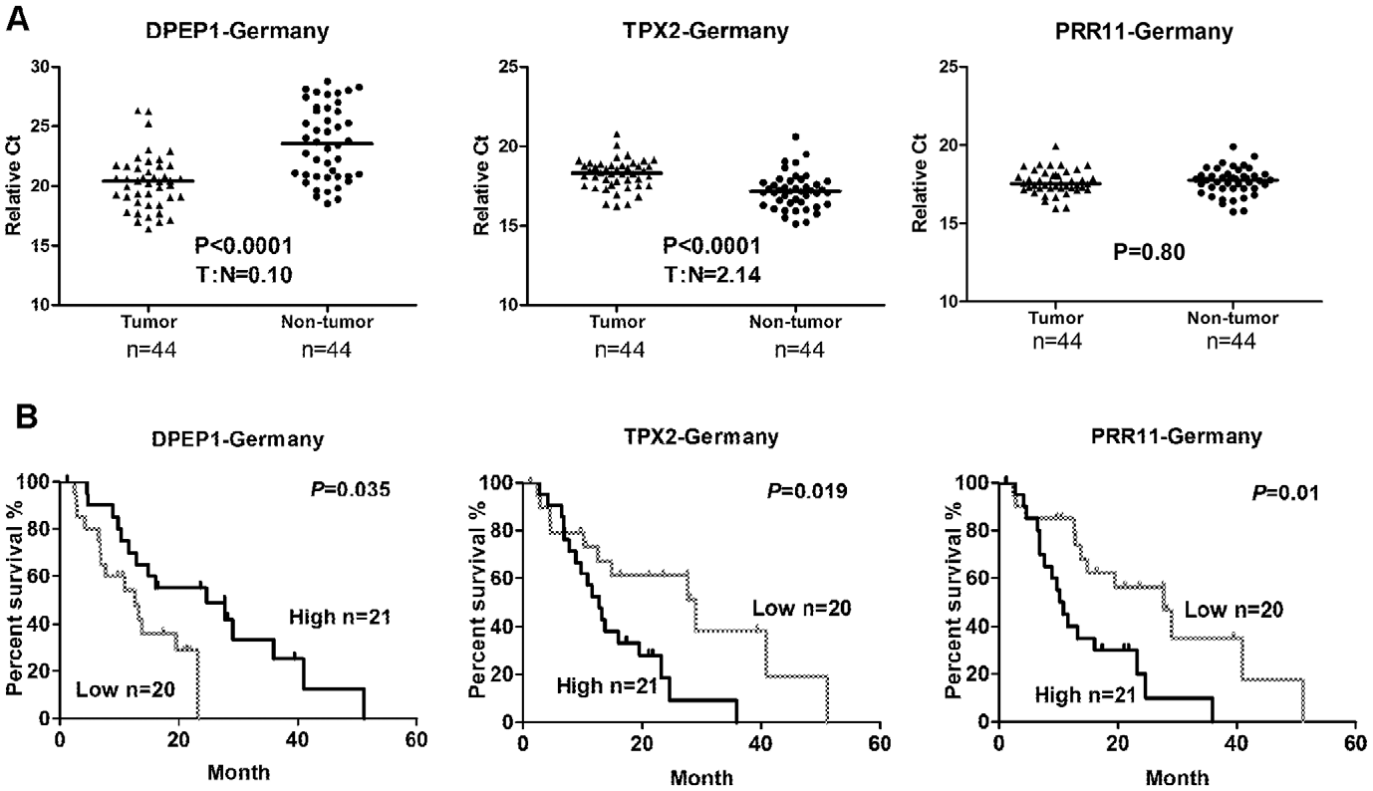
DPEP1, TPX2 and PRR11 expression are associated with cancer-specific death in test Germany cohort. **A:** DPEP1 was expressed at a lower level and TPX2 at a higher level in pancreatic tumors as compared to adjacent non-tumor tissue. Dot plots represent gene expression level with relative threshold cycle value (Ct) normalized with endogenous control gene GAPDH. Bars indicate median value. Wilcoxon matched-pairs t-tests *P* value and tumor: non-tumor ratios (T∶N) are indicated in the graphs. **B:** Kaplan Meier analysis of DPEP1, TPX2, and PRR11 in the Germany test cohort.

The association of gene expression with cancer-specific mortality in Germany test cohort was evaluated with qRT-PCR data in which we dichotomized high and low expression for each gene, consistent with our methods in the context of microarray experiments for the test cohort. We found that a decreased expression of *DPEP1*, or an increased expression of *TPX2* and *PRR11* transcripts, was associated with poor survival (*P*<0.05, Kaplan-Meier log-rank test) in the test cohort (Figure 1B).

**Figure 2:**
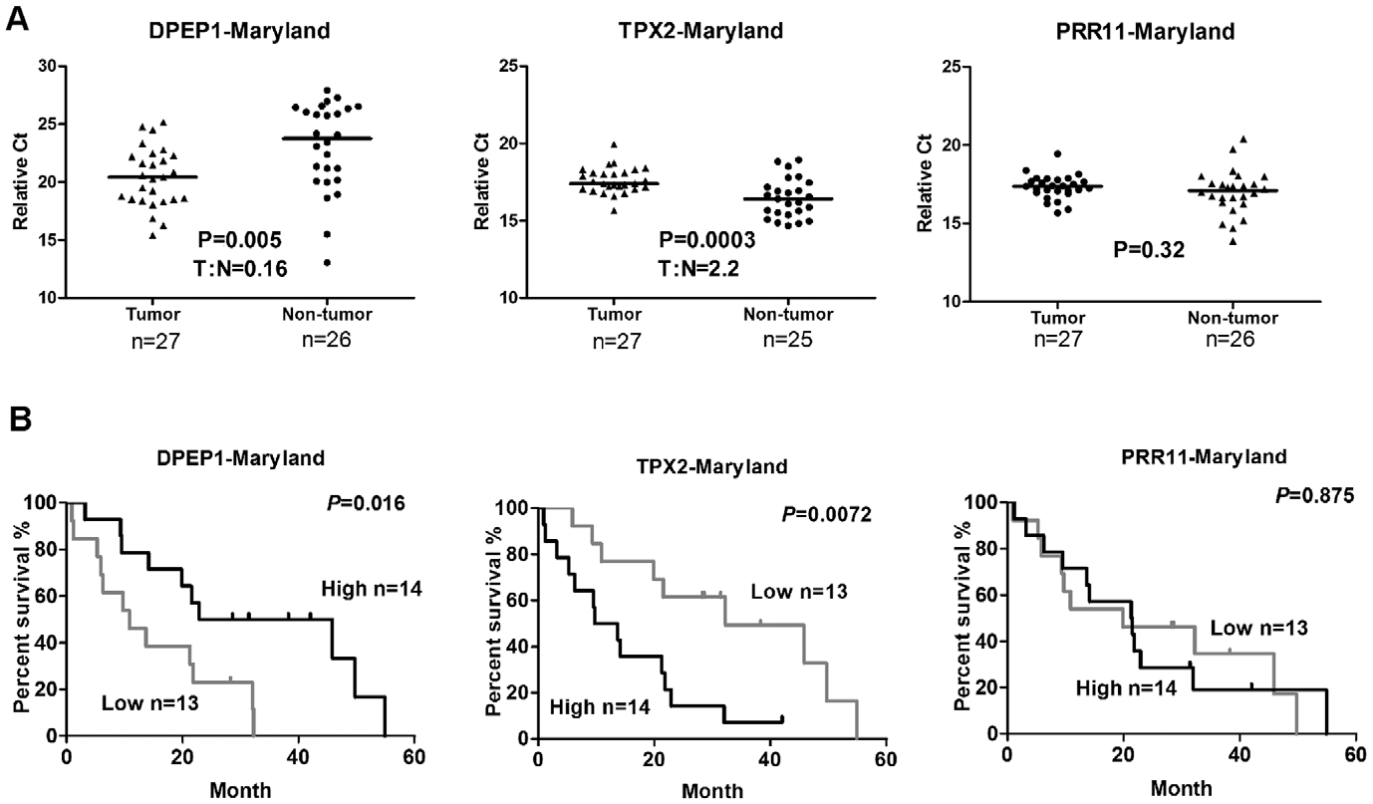
Validation of DPEP1 and TPX2 in independent Maryland cohort. **A:** DPEP1 and TPX2 were differentially expressed in Maryland validation cohort at a similar level as in Germany cohort. Dot plots represent gene expression level with relative threshold cycle value (Ct) normalized with endogenous control gene GAPDH. Bars indicate median value. Wilcoxon matched-pairs t-tests *P* value and tumor: non-tumor ratios (T∶N) are indicated in the graphs. **B:** The association of DPEP1 or TPX2 with patient survival was validated in the Maryland cohort. PRR11 was not associated with survival in the validation cohort.

To validate these findings, we performed qRT-PCR for these 3 genes in an independent Maryland validation cohort and analyzed their associations with cancer-specific mortality. A lower *DPEP1* expression or a higher *TPX2* expression in tumor was associated with poor survival in the Maryland validation cohort (*P* = 0.016 and *P* = 0.007 respectively, Kaplan-Meier log-rank test) (Figure 2B), consistent with our results in the Germany test cohort. *PRR11* expression failed to show statistically significant association with cancer-specific mortality in Maryland validation cohort (Figure 2B, *P* = 0.875).

*DPEP1* expression was decreased (T: N ratio of 0.1 in test cohort and 0.16 in validation cohort, *P*<0.01) and *TPX2* expression was increased (T: N ratio of 2.14 in test cohort and 2.2 in validation cohort, *P*<0.001) in human pancreatic tumor tissues, as compared with surrounding non-tumor tissues in both cohorts (Figure 1, 2). In addition, histological grade was significantly correlated with DPEP1 (Spearman correlation coefficient = −0.35, *P* = 0.004) or TPX2 (Spearman correlation coefficient = 0.44, *P* = 0.0002) gene expression (Table S2).

In order to determine the localization of DPEP1 protein, we performed immunohistochemical staining on paraffin sections, which showed that DPEP1 expresses in both ductal carcinoma cells and stromal fibroblast cells (Figure S2).

### DPEP1 and TPX2 are independent predictors of cancer-specific mortality in PDAC

The Germany and Maryland cohorts were similar in gender, resection margin status, TNM staging and overall survival. Therefore, to increase statistical power, these cohorts were combined for further analyses. Low DPEP1 or high TPX2 expression was associated with poor cancer-specific mortality for all patients in the combined cohort (P<0.01, Kaplan-Meier log rank; Figure S3A). When stratified by resection margin status (positive vs. negative), both DPEP1 and TPX2 were associated with cancer-specific mortality in resection margin positive patients (P<0.01, Kaplan Meier log rank; Figure S3B). Furthermore, TPX2 was also associated with prognosis in resection margin-negative patients (P<0.05, Kaplan Meier log rank; Figure S3C).

Univariate and Multivariate Cox proportional hazards analysis was used to further evaluate the association of DPEP1 or TPX2 expression in tumors with patient outcome in the combined cohort (Table 2). The most commonly used pathologic predictors of survival after surgery are the stage, grade, and the resection margin status. Resection margin status was confirmed as an influential prognostic factor in many studies [13]. In our study, Univariate Cox regression analysis for all cases found that high DPEP1 (hazard ratio (HR), 0.42; 95% CI, 0.24–0.76; *P* = 0.004), high TPX2 (HR, 2.92; 95% CI, 1.57–5.40; *P* = 0.001), and resection margin positive status (HR, 1.77; 95% CI, 0.99–3.18; *P* = 0.05) were each associated with prognosis but not the tumor stage or grade (Table 2). Multivariate analyses showed that both high DPEP1 (HR, 0.51; 95% CI, 0.27–0.96) and high TPX2 (HR, 2.42; 95% CI, 1.27–4.61) were independent of one another and resection margin status. Additionally, the final multivariate model including DPEP1, TPX2 and resection margin did significantly better than a model without TPX2 or DPEP1 (P = 0.012 or P = 0.036 respectively, likelihood ratio test). Therefore, the combination of resection margin status, DPEP1 and TPX2 expression is significantly better at predicting patient outcome in resected PDAC cases than any of these factors alone, demonstrating their clinical significance.

**Table 2:** Cox regression analysis of DPEP1 and TPX2 expression with caner-specific mortality on combined Germany test cohort and Maryland validation cohort.

Variables (comparison/referent)	Univariate analysis^a^		Multivariate analysis^b^	
	HR (95% CI)	*P*	HR (95% CI)	*P*
DPEP1(high/low)	0.43 (0.24–0.76)	0.004	0.51 (0.27–0.94)	0.032
TPX2(high/low)	2.92 (1.57–5.40)	0.001	2.42 (1.27–4.61)	0.007
Resection Margin (R1/R0)	1.77 (0.99–3.18)	0.050	1.90 (1.05–3.45)	0.033
Grading (G3&4/1&2)	1.72 (0.97–3.04)	0.063		
Tumor stage (IIB-IV/I-IIA)	1.58 (0.80–3.13)	0.191		

a Univariate analysis is adjusted for cohort membership only.

b Multivariate analysis is adjusted for cohort membership, TPX2, DPEP1, and resection margin status. Multivariate analysis used stepwise addition and removal of clinical covariates found to be associated with survival in Univariate model and final models include only those covariates that were significantly associated with survival (*P*<0.05).

### Overexpression of DPEP1 inhibits cell invasion in vitro

**Figure 3:**
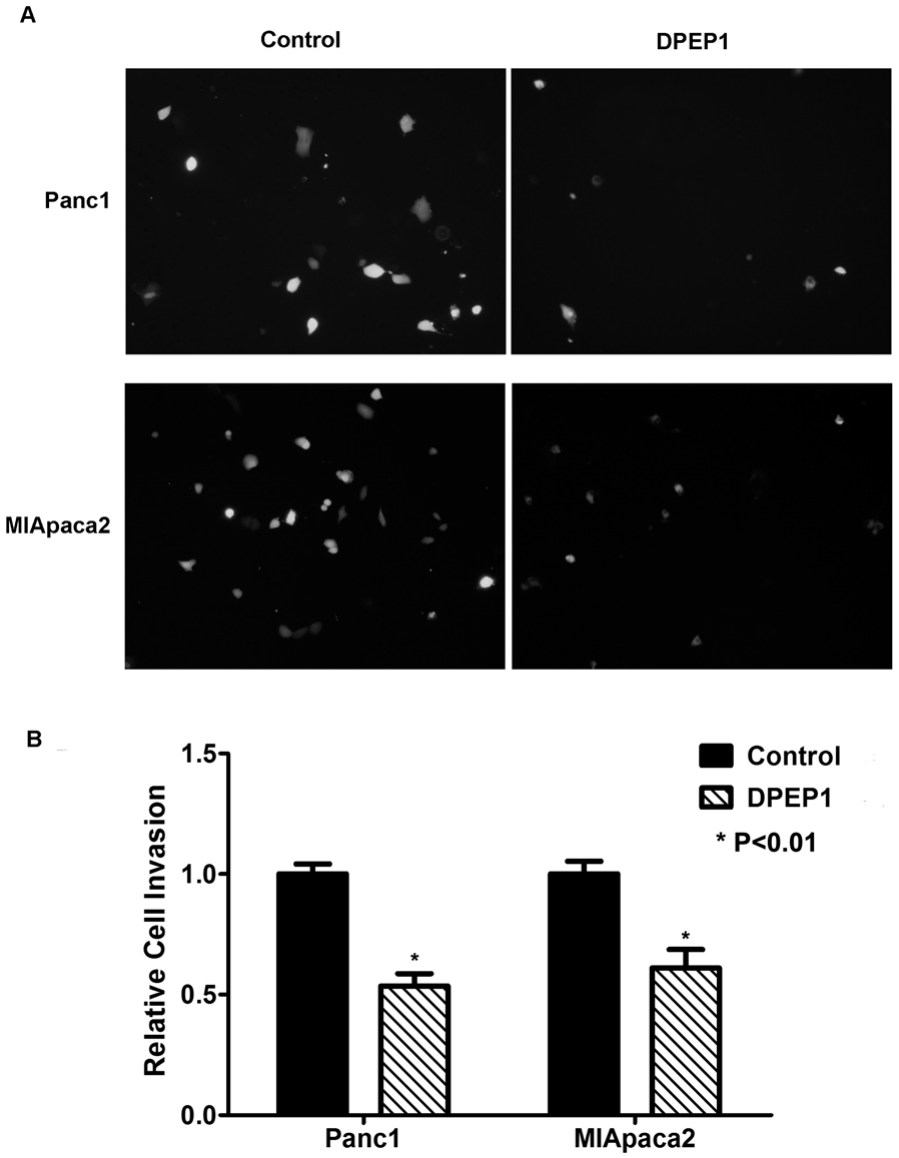
DPEP1 overexpression inhibits cell invasion in Panc1 and MIApaca2 cells. **A:** Cell invasion was analyzed in Panc1 (upper panel) and MIApaca2 (lower panel) cells using Biocoat matrigel invasion assay. The invaded GFP-positive cells were counted under a fluorescence microscope. **B:** Relative cell invasion is expressed as the ratio of the percent invasion of a test cell over the percent invasion of a control cell (* P<0.01).

The oncogenic role of TPX2 in pancreatic cancer development has been demonstrated in previous studies [14]. However, the biological function of DPEP1 in PDAC remains unknown. Therefore, we examined if the expression of DPEP1 affects the cellular growth and invasion of pancreatic cancer cell lines. Cell lines that were transfected with pCMV-*DPEP1*-GFP showed enhanced expression of DPEP1 as compared with control pCMV-GFP transfectants in Panc1 and MIApaca2 respectively (Figure S4 A&B). DPEP1 overexpression had no effect on cell proliferation and colony formation in Panc1 and MIApaca2 cells (Figure S4 C&D). We then compared the invasive ability of DPEP1 cDNA transfectants and control GFP transfectants in Panc1 and MIApaca2 cells using 10% FBS as a chemoattractant in Matrigel invasion assays. DPEP1 overexpressing cells showed markedly decreased invasive ability compared with control cells in both Panc1 and MIApaca2 cells (*P*<0.01, Figure 3).

### DPEP1 sensitizes pancreatic cancer cells to Gemcitabine

**Figure 4:**
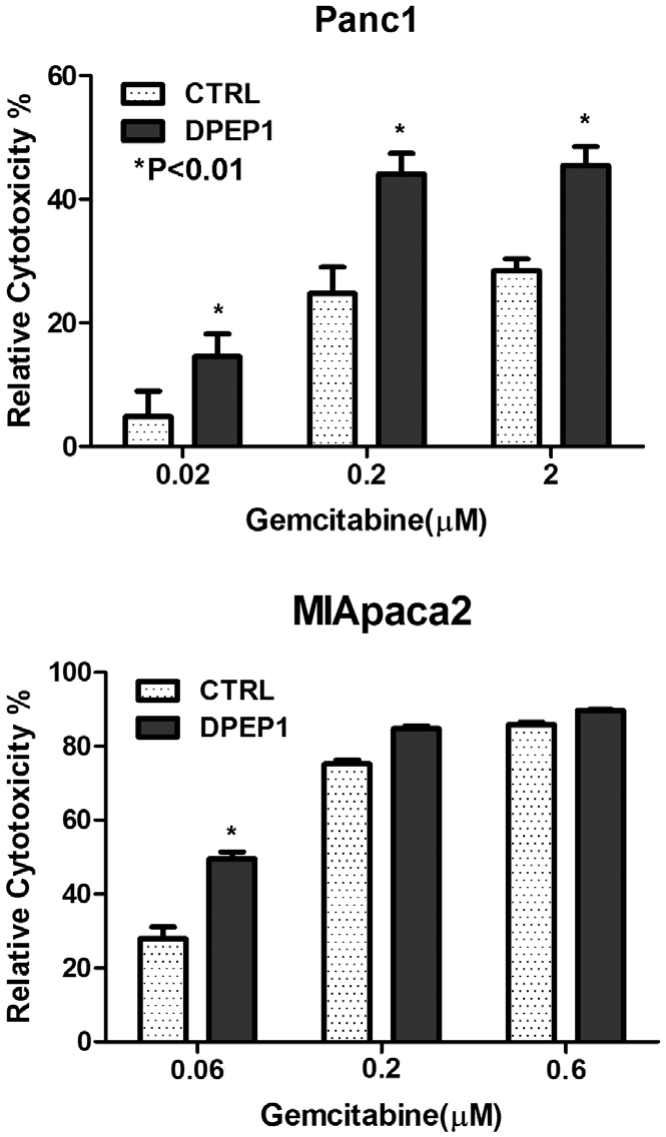
DPEP1 overexpression enhances sensitivity to gemcitabine. DPEP1 overexpressing cells and control cells were analyzed for cellular sensitivity to gemcitabine using Panc1 (A) and MIApaca2 (B). Overexpession of DPEP1 increased the sensitivity of Panc1 and MIApaca2 cells to gemcitabine. Control cells are Panc1 or MIApaca2 cells transfected with GFP control vectors. Cells were treated with Gemcitabine for 96 hours at different doses. The MTS assay was used to quantitate cytotoxicity (cell death) according to the manufacturer's instructions. Relative cytotoxicity (%) was calculated using the formula: [1−(OD_570_ of drug treated cells/OD_570_ of untreated cells)]×100%. Data are means ± S.D. from 3 independent experiments. * T-test *P*<0.01.

Rapid development of resistance in PDAC inevitably translates into poor patient outcomes [15]. To determine the effect of DPEP1 expression on the sensitivity of pancreatic cancer cell lines to gemcitabine, we examined cytotoxicity of gemcitabine on Panc1 and MIApaca2 cells after 96 h of drug exposure. We found that the relative cytotoxicity to gemcitabine at 0.2 µM for GFP control and DPEP1 transfectants were 25% and 44% respectively in Panc1 cells (Figure 4A, *P*<0.01). The sensitivity of MIApaca2 cells to lower dose of gemcitabine (0.06 µM) is also increased by DPEP1 overexpression as compared to control cells (50% vs 28% of cytotoxicity, Figure 4B). These results demonstrated that DPEP1 overexpression significantly increased the sensitivity to gemcitabine (P<0.01) in pancreatic cancer cells.

### EGF/MEK/ERK signaling is involved in regulating DPEP1 expression

Growth factor EGF can activate RAS-RAF-MEK-ERK as well as PI3K pathways [16]. High level of EGF and constitutive activation of the RAS-RAF-MEK-ERK pathway was found in pancreatic cancer. To investigate the possible mechanism for the regulation of DPEP1 expression in pancreatic cancer, we examined the levels of DPEP1 in Panc1 cells in response to EGF (30 nM), MEK1/2 inhibitor AZD6244 (1.5 µM) and PI3K inhibitor LY294002 (1.5 µM).

**Figure 5:**
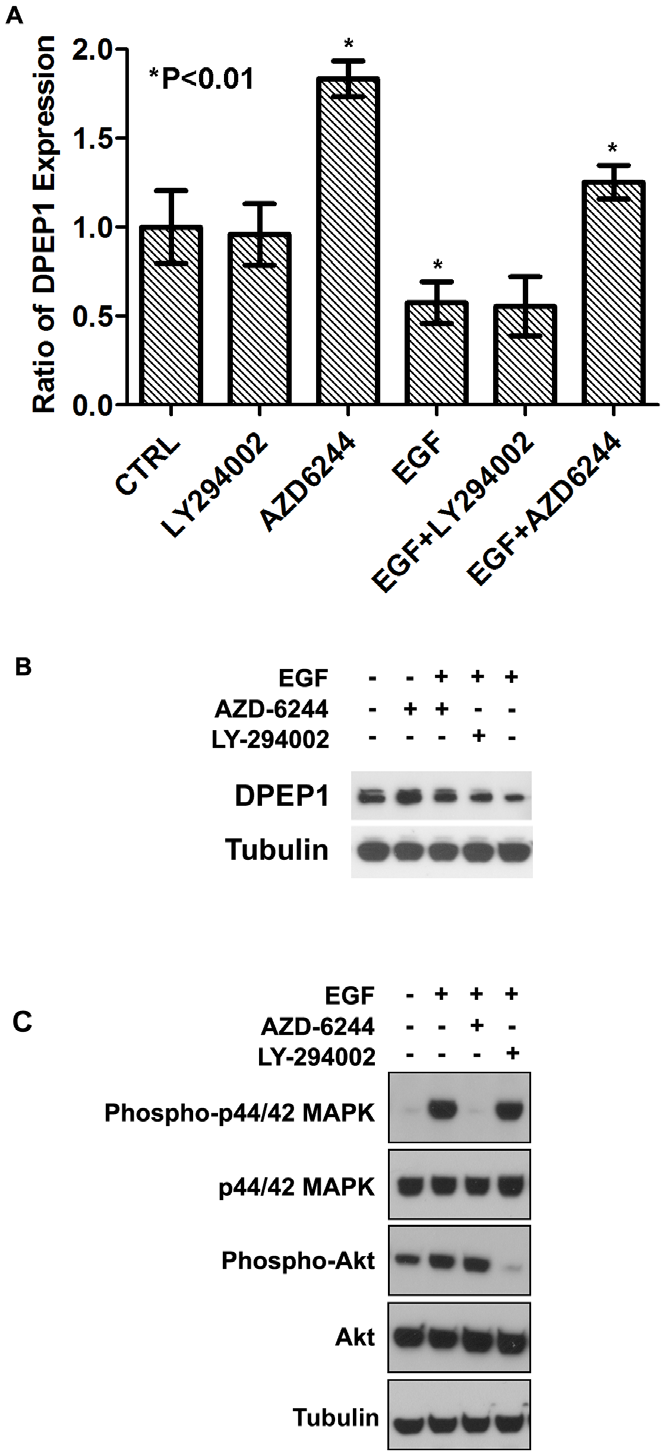
Effect of EGF, AZD6244 and LY294002 on DPEP1 expression. Panc1 cells were starved in 0.1% FBS for 16 hours before treatments. Cells were treated with RPMI medium containing EGF (30 ng/mL), AZD6244 (1.5 µM) or LY294002 (1.5 µM) alone for 24 hours. EGF+AZD6244 or EGF+LY294002: Panc1 ells were pretreated with AZD6244 or LY294002 for 1 h prior to the addition of EGF. Untreated control cells were maintained in RPMI with DMSO. **A:** Real-time PCR was done to determine DPEP1 mRNA levels. Ratio of DPEP1expression represents the effect of treatment on gene expression compared to untreated control. Data are means ± S.D. from 3 independent experiments. * T-test *P*<0.01. **B:** Western blot showed similar changes at protein level of DPEP1 after 24 hour treatment. **C:** Western blot demonstrated the efficiency and specificity of AZD6244 and LY294002.

Treatment with EGF significantly decreased DPEP1 by about 50% (P<0.01). The inhibition of DPEP1 expression by EGF was rescued by AZD6244 (MEK1/2 inhibitor) but not LY294002 (PI3K inhibitor) (Figure 5A and B). A significant increase (∼2 fold) in DPEP1 gene expression was also found when cells were treated with AZD6244 alone (P<0.01) in 24 h (Figure 5A and Figure S5). However, LY294002 alone had no effect on DPEP1 expression. Western blot demonstrated the efficiency and specificity of AZD6244 and LY294002 (Figure 5C). These data indicate that MEK/ERK pathway but not PI3K pathway is involved in regulating DPEP1 expression in pancreatic cancer.

## Discussion

To identify biologically-relevant genes with therapeutic potential for PDAC, we first identified genes that were associated with cancer-specific mortality by microarray gene expression analysis and validated them by qRT-PCR in two independent cohorts of resected PDAC cases. We demonstrated that low DPEP1 expression is significantly associated with poor survival in the Germany test cohort and the Maryland validation cohort. Multivariate analysis showed that the association of DPEP1 with cancer-specific mortality was independent of resection margin status. We also found a negative correlation of DPEP1 gene expression with histological grade (Spearman correlation coefficient = −0.35, *P* = 0.004). To our knowledge, our data for the first time suggested that DPEP1 may be a useful prognostic indicator, in addition to resection margin status, for PDAC patients following resection. Our study showed a marked reduction in DPEP1 expression in pancreatic tumors as compared with non-tumor tissues using both microarray and qPCR, which is consistent with the ONCOMINE data mining [17]. Earlier reports have provided evidence for altered expression of DPEP1 in various malignancies [18], [19]. Loss of DPEP1 expression was associated with breast cancer and Wilms' tumor [20], [21]. However, DPEP1 is highly expressed in colon tumors compared to matched normal mucosa [22]. In addition to DPEP1, we also found that higher TPX2 expression is associated with poor outcome in two independent cohorts of resected PDAC patients, supporting the oncogenic role of TPX2 in several solid tumors including pancreatic adenocarcinoma [23], [24].

The fact that a higher DPEP1 expression level is associated with better patient outcome indicated its possible inhibitory role in tumor aggressiveness. In the present study, we have elucidated the function of DPEP1 in pancreatic cancer cells. Our data showed that DPEP1 overexpression in pancreatic cancer cell lines significantly inhibits cell invasion (Figure 3) but has no effect on cell proliferation and tumor colony formation (Figure S4 C and D). Moreover, DPEP1 over-expression increases the sensitivity of pancreatic cancer cells to chemotherapeutic drug gemcitabine. The molecular mechanism by which DPEP1 inhibits tumor progression and aggressiveness is not known. Interestingly, immunostaining of archival human pancreatic cancer tissues showed DPEP1 expression in both carcinoma cells and peritumoral stroma. Future studies will investigate whether DPEP1 in stromal cells plays a role in pancreatic cancer development. DPEP1 is also implicated in the metabolism of glutathione, an important antioxidant [20], [21], which helps in maintaining the optimal redox state in the cellular microenvironment and protect cells against pathological stress. Reduced DPEP1 expression leads to dysregulation of glutathione homeostasis, which may promote tumorigenesis [25].

Given the association of high DPEP1 with a better outcome in pancreatic cancer and its potential role in inhibiting tumor aggressiveness, a logical hypothesis is that a treatment, which can increase DPEP1, may improve patients' survival. In this study we found that EGF/RAS/MEK pathway is involved in regulating DPEP1 expression. Inhibition of MEK/ERK pathway by AZD6244 increases DPEP1 level in Panc1 cells. AZD6244 is a potent and selective MEK inhibitor that has been selected for clinical development because of its potency and favorable pharmacokinetic profile [26]. Further delineation of pathways that regulate DPEP1 expression in pancreatic cancer may provide insights into the genes and pathways associated with DPEP1 and facilitate the development of more effective therapeutic strategies for PDAC.

In conclusion, we showed for the first time that DPEP1 is a significant predictor for patient outcome in resected PDAC cases. Functional evidence that DPEP1 inhibits cancer cell invasion and enhances sensitivity to gemcitabine, suggests DPEP1 as a candidate target for designing therapeutic strategies.

## Supporting Information

Figure S1 **A:** Network analysis was preformed to provide a graphical representation of 36 genes selected from microarray analysis using IPA. Genes in this top network are associated with cancer, cell cycle, and cellular movement. Green icons indicate downregulated genes and red indicates upregulated genes. P value and fold change of gene expression comparison were labeled under each gene symbol. **B:** Correlation of the tumor vs. nontumor tissue expression ratios, comparing the qRT-PCR data with microarray data in the Germany test cohort. Human GAPDH was used as endogenous control for qRT-PCR to normalize across the samples. Spearman correlation test r = 0.89, P<0.0001.
(TIF)

Figure S2 **Representative immunostaining of different levels of DPEP1 expression in two primary PDAC archived samples from tissue microarray slides.**
(TIF)

Figure S3 **Combined analysis of Germany test and Maryland validation cohorts.** Germany test and Maryland validation cohorts were combined together to increase statistical power. **A:** Kaplan Meier analysis of DPEP1 and TPX2 in combined cohort. **B:** Kaplan Meier analysis of DPEP1 and TPX2 stratified by resection margin status. R1: patients with positive microscopic resection margins; R0: patients with negative resection margins.
(TIF)

Figure S4 **Effect of DPEP1 on pancreatic cancer cell proliferation and colony formation.** Cell proliferation of Panc1 and MIApaca2 cells monitored by MTT assay from day 2 to day 10. Increased expression of DPEP1 in transfected cells were demonstrated by quantitative RT-PCR (**A**) and western blot (**B**). There was no significant difference between DPEP1 overexpressing cells and control cells (P>0.1) in cell growth (**C**) and colony formation (**D**).
(TIF)

Figure S5 **DPEP1 mRNA level at different time points (6 h, 12 h, 24 h and 48 h) after adding AZD6244.** Real-time PCR was done to determine DPEP1 mRNA levels. Relative expression of DPEP1 represents the effect of treatment on gene expression compared to untreated control. Data are means ± S.D. from 3 independent experiments. * T-test *P*<0.01.
(TIF)

Table S1 **A list of 36 genes selected from microarray analysis and evaluated by RT-PCR in Germany test cohort.**
(DOC)

Table S2 **Correlation of histological grade, stage or resection margin with DPEP1 or TPX2 gene expression.**
(DOC)
