# Supplementary figures and images for: DPEP1 Inhibits Tumor Cell Invasiveness, Enhances Chemosensitivity and Predicts Clinical Outcome in Pancreatic Ductal Adenocarcinoma
Source: PLoS One. 2012 Feb 20;7(2):e31507. doi: 10.1371/journal.pone.0031507 (PMC3282755; doi:10.1371/journal.pone.0031507)

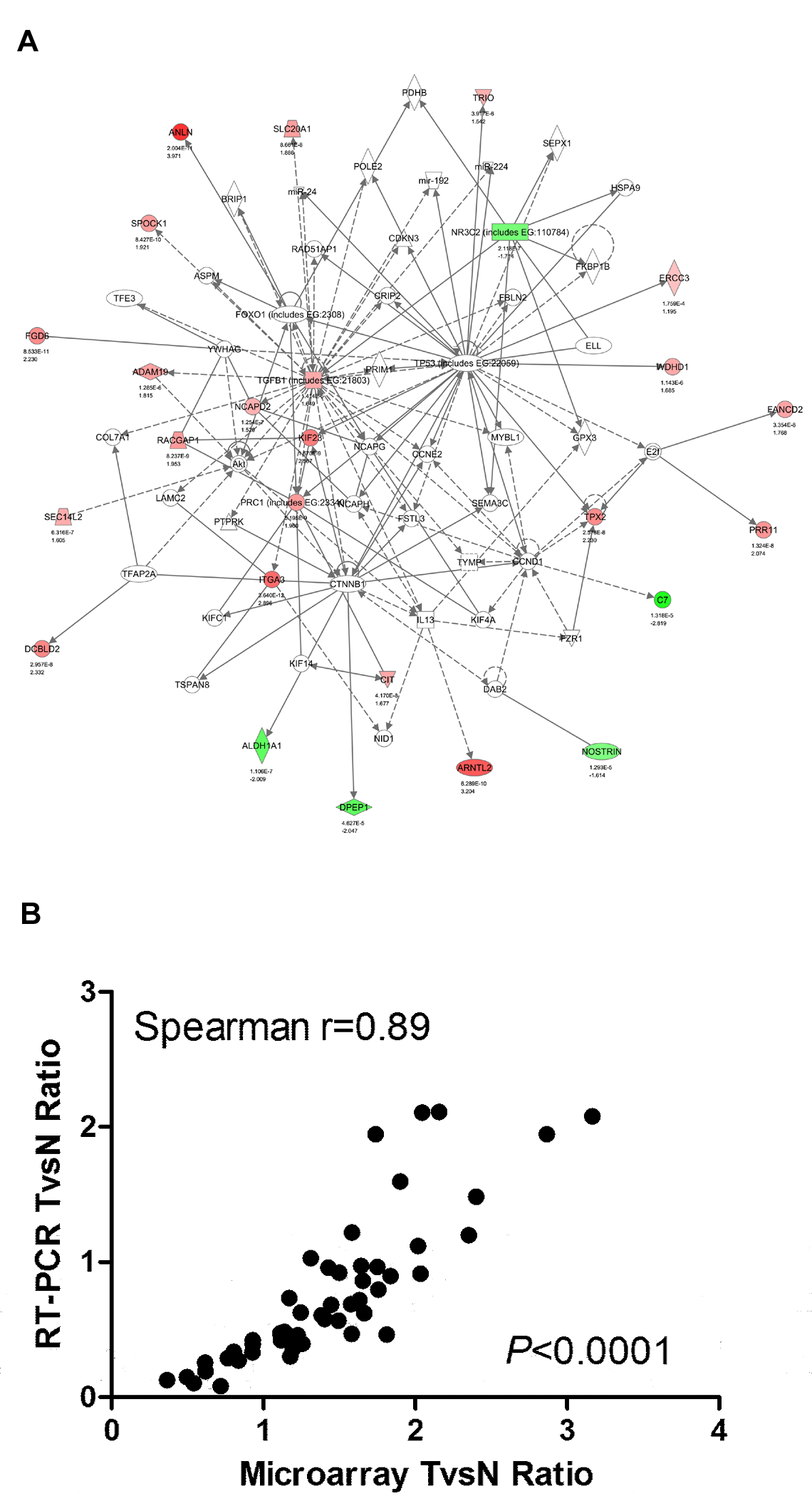

Supplement: Figure S1 — A: Network analysis was preformed to provide a graphical representation of 36 genes selected from microarray analysis using IPA. Genes in this top network are associated with cancer, cell cycle, and cellular movement. Green icons indicate downregulated genes and red indicates upregulated genes. P value and fold change of gene expression comparison were labeled under each gene symbol. B: Correlation of the tumor vs. nontumor tissue expression ratios, comparing the qRT-PCR data with microarray data in the Germany test cohort. Human GAPDH was used as endogenous control for qRT-PCR to normalize across the samples. Spearman correlation test r = 0.89, P<0.0001. (TIF) [file pone.0031507.s001.tif]

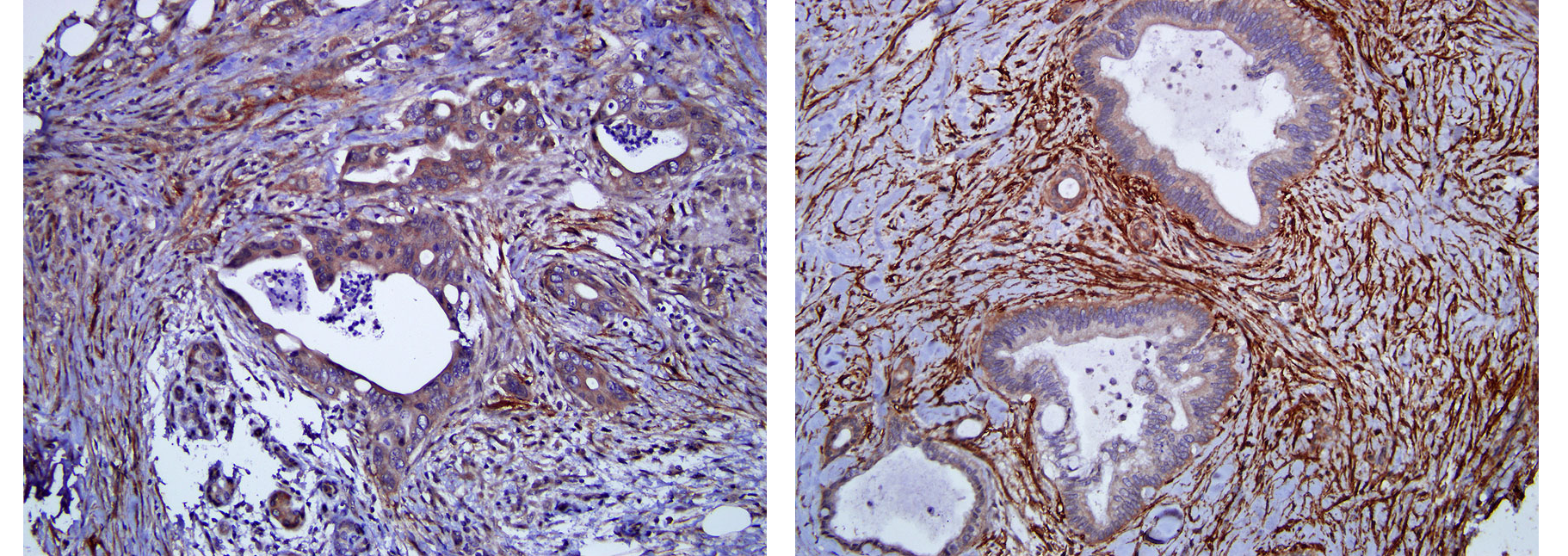

Supplement: Figure S2 — Representative immunostaining of different levels of DPEP1 expression in two primary PDAC archived samples from tissue microarray slides. (TIF) [file pone.0031507.s002.tif]

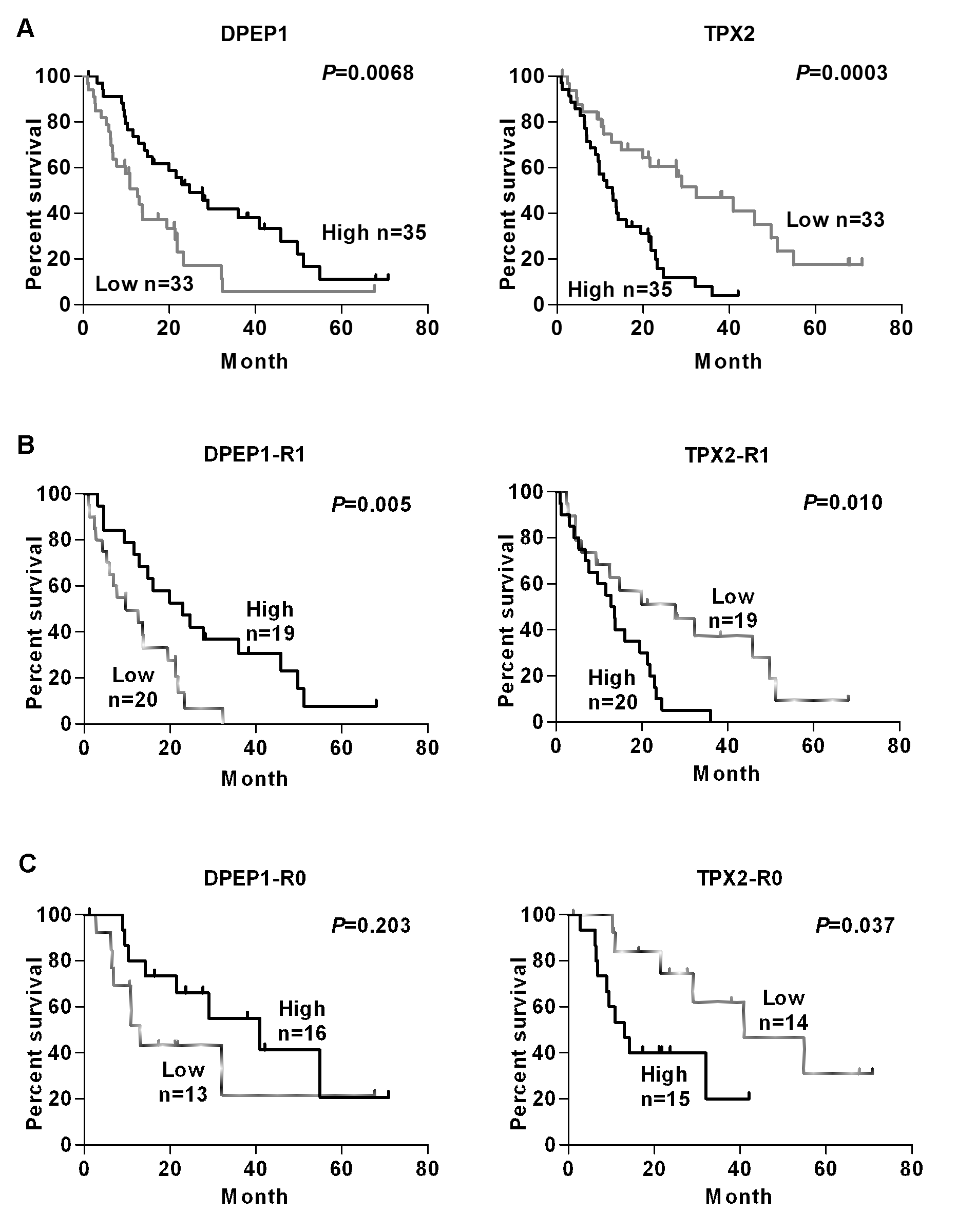

Supplement: Figure S3 — Combined analysis of Germany test and Maryland validation cohorts. Germany test and Maryland validation cohorts were combined together to increase statistical power. A: Kaplan Meier analysis of DPEP1 and TPX2 in combined cohort. B: Kaplan Meier analysis of DPEP1 and TPX2 stratified by resection margin status. R1: patients with positive microscopic resection margins; R0: patients with negative resection margins. (TIF) [file pone.0031507.s003.tif]

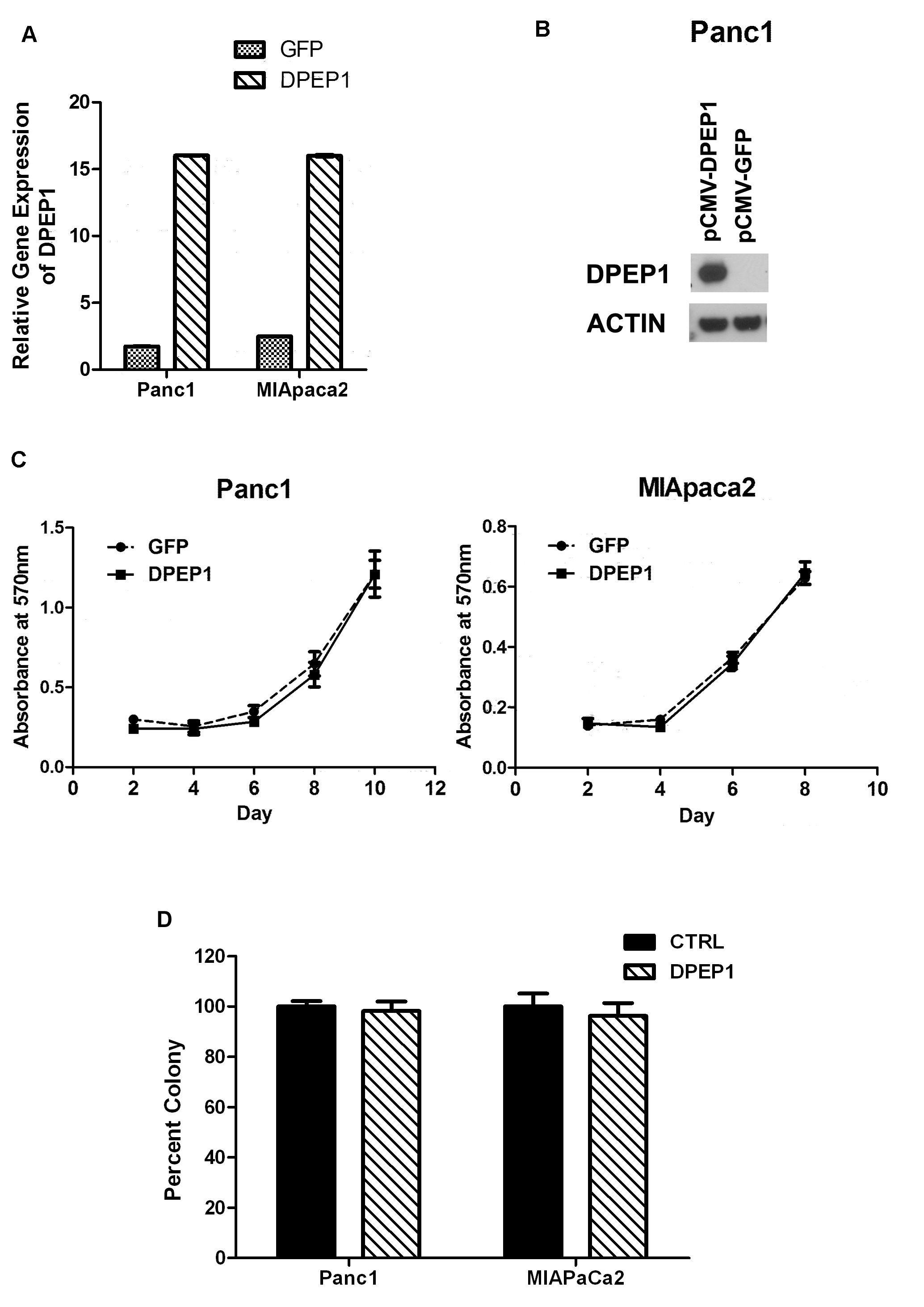

Supplement: Figure S4 — Effect of DPEP1 on pancreatic cancer cell proliferation and colony formation. Cell proliferation of Panc1 and MIApaca2 cells monitored by MTT assay from day 2 to day 10. Increased expression of DPEP1 in transfected cells were demonstrated by quantitative RT-PCR (A) and western blot (B). There was no significant difference between DPEP1 overexpressing cells and control cells (P>0.1) in cell growth (C) and colony formation (D). (TIF) [file pone.0031507.s004.tif]

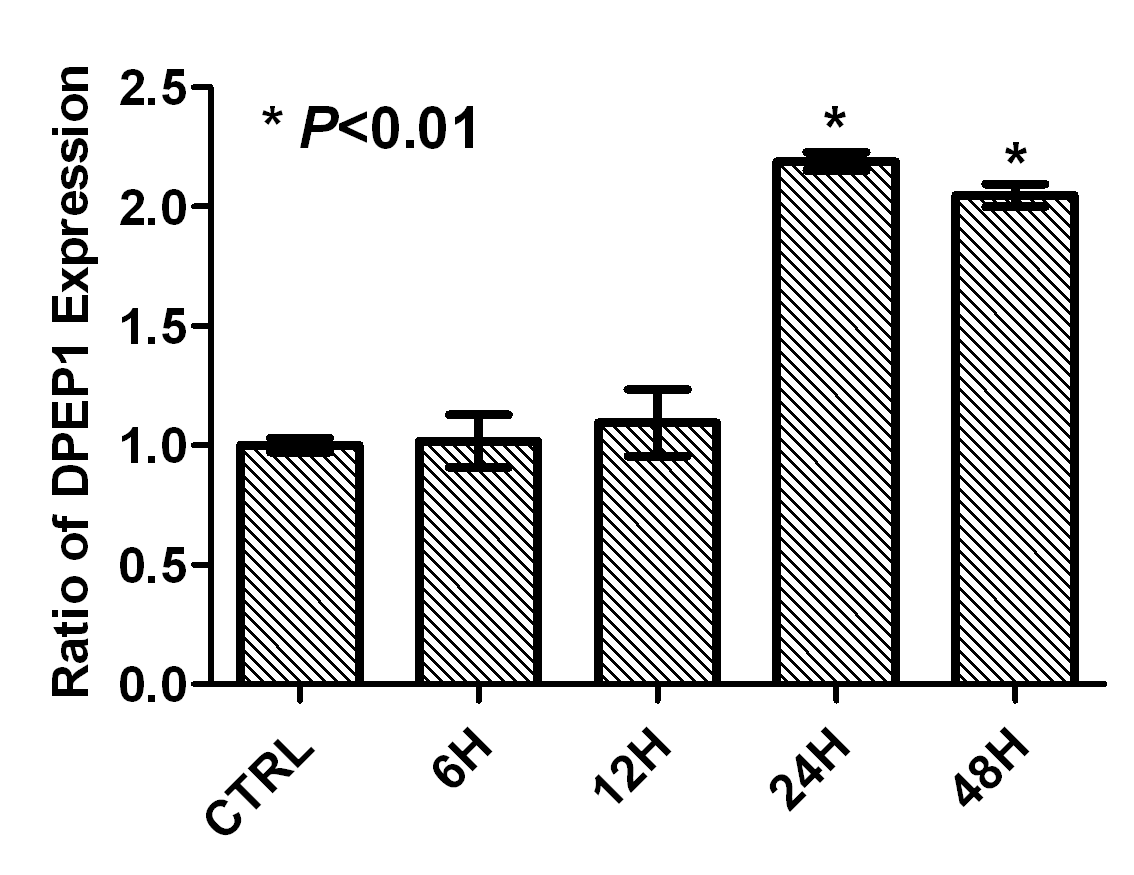

Supplement: Figure S5 — DPEP1 mRNA level at different time points (6 h, 12 h, 24 h and 48 h) after adding AZD6244. Real-time PCR was done to determine DPEP1 mRNA levels. Relative expression of DPEP1 represents the effect of treatment on gene expression compared to untreated control. Data are means ± S.D. from 3 independent experiments. * T-test P<0.01. (TIF) [file pone.0031507.s005.tif]
