# Supplementary material for: DPEP1 Inhibits Tumor Cell Invasiveness, Enhances Chemosensitivity and Predicts Clinical Outcome in Pancreatic Ductal Adenocarcinoma
Source: PLoS One. 2012 Feb 20;7(2):e31507. doi: 10.1371/journal.pone.0031507 (PMC3282755; doi:10.1371/journal.pone.0031507)
Supplement: Table S1 — A list of 36 genes selected from microarray analysis and evaluated by RT-PCR in Germany test cohort. (DOC) [file pone.0031507.s006.doc]

**Table S1. A list of 36 genes selected from microarray analysis and evaluated by RT-PCR in Germany test cohort.**

|  |  |  | **RT-PCR** | | **Microarray analysis** | | | |
| --- | --- | --- | --- | --- | --- | --- | --- | --- |
| **Gene Symbol** | **RefSeq** | **Taqman assay ID** | **T vs.N Ratio** | **T vs.N *p*-value** | **T vs.N Ratio** | **T vs.N *p*-value** | **Hazard Ratio** | **Cox**  ***p*-value** |
| ADAM19 | NM_033274 | Hs00224960_m1 | 1.9 | 6.6E-05 | 1.8 | 1.3E-06 | 2.1 | 0.043 |
| ADCY7 | NM_001114 | Hs00936808_m1 | 1.0 | 7.8E-01 | 1.4 | 2.1E-05 | 3.2 | 0.022 |
| ALDH1A1 | NM_000689 | Hs00946916_m1 | 0.1 | 8.1E-12 | 0.5 | 1.1E-07 | 0.6 | 0.018 |
| ANLN | NM_018685 | Hs01122612_m1 | 2.1 | 1.3E-05 | 4.0 | 2.0E-11 | 1.6 | 0.008 |
| ARNTL2 | NM_020183 | Hs00368068_m1 | 1.5 | 9.6E-04 | 3.2 | 6.3E-10 | 1.4 | 0.044 |
| C7 | NM_000587 | Hs00175109_m1 | 0.1 | 4.7E-09 | 0.4 | 1.3E-05 | 0.7 | 0.006 |
| CAPRIN2 | NM_001002259 | Hs00225069_m1 | 0.6 | 3.8E-05 | 1.6 | 1.6E-06 | 1.9 | 0.035 |
| CDO1 | NM_001801 | Hs00156447_m1 | 0.1 | 9.4E-09 | 0.7 | 5.2E-08 | 0.4 | 0.031 |
| CIT | NM_007174 | Hs00392339_m1 | 0.9 | 4.3E-01 | 1.7 | 4.2E-08 | 3.6 | 0.000 |
| DCBLD2 | NM_080927 | Hs00294635_m1 | 2.1 | 2.9E-03 | 2.3 | 3.0E-08 | 1.7 | 0.011 |
| DPEP1 | NM_004413 | Hs01116752_m1 | 0.1 | 2.4E-07 | 0.5 | 4.6E-05 | 0.6 | 0.043 |
| ERCC3 | NM_000122 | Hs01554450_m1 | 0.5 | 1.8E-09 | 1.2 | 1.8E-04 | 11.1 | 0.016 |
| FANCD2 | NM_033084 | Hs00276992_m1 | 0.7 | 6.4E-03 | 1.8 | 3.4E-08 | 2.3 | 0.026 |
| FANCI | NM_001113378 | Hs00383049_m1 | 0.9 | 1.3E-01 | 1.8 | 5.8E-08 | 2.1 | 0.023 |
| FGD6 | NM_018351 | Hs00217947_m1 | 1.2 | 1.1E-01 | 2.2 | 8.5E-11 | 1.7 | 0.050 |
| ITGA3 | NM_002204 | Hs00233722_m1 | 1.9 | 1.2E-06 | 2.9 | 3.6E-12 | 1.6 | 0.035 |
| KIF23 | NM_138555 | Hs00370852_m1 | 0.9 | 4.9E-01 | 2.6 | 1.6E-09 | 2.0 | 0.003 |
| KNTC1 | NM_014708 | Hs00206854_m1 | 0.7 | 6.5E-05 | 1.6 | 7.8E-07 | 2.4 | 0.015 |
| NCAPD2 | NM_014865 | Hs00274505_m1 | 0.6 | 1.6E-06 | 1.5 | 1.3E-07 | 2.7 | 0.021 |
| NOSTRIN | NM_001039724 | Hs00976555_m1 | 0.3 | 7.0E-10 | 0.6 | 1.3E-05 | 0.4 | 0.003 |
| NR3C2 | NM_000901 | Hs01031809_m1 | 0.2 | 5.6E-11 | 0.6 | 2.1E-07 | 0.5 | 0.019 |
| PAFAH1B2 | NM_002572 | Hs00733118_m1 | 0.4 | 2.5E-06 | 1.2 | 9.0E-06 | 4.9 | 0.050 |
| PRC1 | NM_003981 | Hs00187740_m1 | 0.8 | 1.2E-01 | 2.0 | 5.2E-09 | 2.2 | 0.007 |
| PRR11 | NM_018304 | Hs00383634_m1 | 1.0 | 8.0E-01 | 2.1 | 1.3E-08 | 2.1 | 0.005 |
| RACGAP1* | NM_013277 | Hs01100049_mH | NA | NA | 2.0 | 8.2E-09 | 3.1 | 0.005 |
| RALGAPB | NM_020336 | Hs00384265_m1 | 0.4 | 3.1E-09 | 1.1 | 7.4E-05 | 11.1 | 0.042 |
| SEC14L2 | NM_012429 | Hs00202756_m1 | 1.2 | 1.2E-01 | 1.6 | 6.3E-07 | 2.2 | 0.029 |
| SEMA3A | NM_006080 | Hs00173810_m1 | 0.5 | 9.5E-05 | 1.8 | 8.0E-05 | 1.8 | 0.035 |
| SLC20A1 | NM_005415 | Hs00965587_m1 | 1.0 | 7.0E-01 | 1.9 | 8.7E-08 | 2.0 | 0.018 |
| SPOCK1 | NM_004598 | Hs00270274_m1 | 1.6 | 2.5E-03 | 1.9 | 8.4E-10 | 2.5 | 0.005 |
| TGFB1 | NM_000660 | Hs00998133_m1 | 1.0 | 6.0E-01 | 1.6 | 1.4E-05 | 2.2 | 0.049 |
| TMEM194A | NM_001130963 | Hs00322591_m1 | 0.4 | 7.8E-09 | 1.4 | 1.3E-05 | 3.2 | 0.009 |
| TPX2 | NM_012112 | Hs00201616_m1 | 2.1 | 4.7E-06 | 2.2 | 2.6E-08 | 2.1 | 0.001 |
| TRIO | NM_007118 | Hs00179276_m1 | 0.5 | 1.8E-09 | 1.5 | 3.9E-06 | 5.9 | 0.004 |
| WDHD1 | NM_007086 | Hs00173172_m1 | 0.7 | 1.2E-05 | 1.7 | 1.1E-06 | 2.9 | 0.001 |
| YEATS2 | NM_018023 | Hs00216001_m1 | 0.6 | 2.9E-06 | 1.5 | 9.5E-08 | 3.6 | 0.009 |

* For RACGAP1, samples with a cycle value greater than 36 were considered of poor quality and removed.
