# Supplementary material for: DPEP1 Inhibits Tumor Cell Invasiveness, Enhances Chemosensitivity and Predicts Clinical Outcome in Pancreatic Ductal Adenocarcinoma
Source: PLoS One. 2012 Feb 20;7(2):e31507. doi: 10.1371/journal.pone.0031507 (PMC3282755; doi:10.1371/journal.pone.0031507)
Supplement: Table S2 — Correlation of histological grade, stage or resection margin with DPEP1 or TPX2 gene expression. (DOC) [file pone.0031507.s007.doc]

**Table S2. Correlation of histological grade, stage or resection margin with DPEP1 or TPX2 gene expression*.**

|  | **DPEP1** | **TPX2** |
| --- | --- | --- |
| **Grading** | | |
| Correlation coefficient | **-0.35** | **0.44** |
| *P* value | **0.004** | **0.0002** |
| **TNM stage** | | |
| Correlation coefficient | -0.07 | 0.18 |
| *P* value | 0.577 | 0.145 |
| **Resection margin** | | |
| Correlation coefficient | -0.19 | 0.02 |
| *P* value | 0.121 | 0.895 |

* Spearman correlation analysis were performed using Stata 11 (StataCorp LP, College Station, Texas)
